# Integrative GWAS and transcriptomic analyses reveal regulatory genes controlling shoot branching in sunflower

**DOI:** 10.3389/fpls.2025.1674383

**Published:** 2025-09-22

**Authors:** Yiyi Sun, Yanwen Wang, Jingyan Bai, Jiatong Guo, Guiting Li, Xiaojie Yang, Qiuzhen Tian, Yingying Huang, Shuping Lv, Hengchun Cao, Lingyun Liu

**Affiliations:** ^1^ School of Life Sciences, Henan University, Kaifeng, Henan, China; ^2^ Henan Key Laboratory of Specific Crops Genomics (Henan Sesame Research Center, Henan Academy of Agricultural Sciences), Zhengzhou, Henan, China; ^3^ Henan Joint Key Laboratory of Specific Oilseed Crops, Zhengzhou, Henan, China; ^4^ Henan Sesame Research Center, Henan Academy of Agricultural Sciences, Zhengzhou, Henan, China; ^5^ Economic Crop Research Institute, Henan Academy of Agricultural Sciences, Zhengzhou, Henan, China

**Keywords:** sunflower, shoot branching, plant architecture, GWAS, transcriptome analysis

## Abstract

Shoot branching plays a crucial role in shaping plant architecture, influencing both yield potential and ornamental qualities in sunflower (*Helianthus annuus* L.), but the genetic mechanisms underlying this trait are still not well understood. To investigate this, we performed a genome-wide association study (GWAS) using 82 sunflower accessions with diverse branching phenotypes and identified 62 significant single-nucleotide polymorphisms (SNPs). Of these, sixty SNPs clustered within a 12.40–17.13 Mb region on chromosome 10. Linkage disequilibrium (LD) block analysis delineated this region, containing 113 genes. Integration of transcriptomic analysis of shoot apical meristems of both branched and unbranched lines revealed 12 differentially expressed genes (DEGs) within the GWAS interval, including two long non-coding RNAs (lncRNAs) that demonstrated significant co-expression with several protein-coding candidates. Notably, one gene (*HanXRQr2_Chr10g0423211*) harbored a nonsynonymous SNP and displayed moderate differences in expression. Tissue-specific RNA-seq and qRT-PCR confirmed the involvement of these 13 genes in branching regulation. Overall, this study advances our understanding of the genetic mechanisms controlling shoot branching in sunflower and highlights candidate genes for targeted breeding to enhance plant architecture.

## Introduction

Sunflower (*Helianthus annuus* L.), belonging to the *Asteraceae* family, is one of the world’s major oilseed crops (Badouin et al., 2017). It is prized for its high levels of unsaturated fatty acids, vitamin E, and protein (Adeleke and Babalola, 2020). Besides its agricultural importance, sunflower is also widely cultivated as an ornamental plant for cut flowers and potted displays. Through domestication, sunflower has undergone substantial morphological changes, evolving from its wild, multi-branched progenitors to predominantly single-stemmed cultivated varieties (Baute et al., 2015; Park and Burke, 2020). While the single-stem architecture improves capitulum size and boosts yield, branched phenotypes are advantageous for their prolonged flowering period (Bachlava et al., 2010; Meena et al., 2025). This trait is especially valuable in hybrid seed production, where branched male lines help reduce flowering asynchrony with single-stemmed female lines. Shoot branching, a fundamental determinant of plant architecture, regulates plant structure through the initiation of axillary meristem (AM) and the growth of axillary buds (Teichmann and Muhr, 2015; Luo et al., 2021). In crop species, shoot branching patterns influence critical agronomic traits, including light interception, planting density adaptability, and ultimately yield (Wang et al., 2018; Guo et al., 2020). Consequently, optimizing the branching patterns is key to enhancing plant productivity and breeding efficiency in species like sunflower. Uncovering the genetic mechanisms that control branching architecture by identifying key regulatory genes offers significant potential for precision breeding in sunflower.

Shoot branching plays a vital role in shaping plant architecture and influencing crop yield. It is regulated by a dynamic interplay between endogenous hormonal signals and exogenous environmental cues (McSteen and Leyser, 2005; Liu et al., 2024). A key aspect of this regulation is the antagonistic interaction between hormones: cytokinin (CK) stimulates bud growth, while auxins, which are directionally transported polarly via PIN proteins, strongly inhibit it, establishing apical dominance (Umehara et al., 2008; Domagalska and Leyser, 2011; Mazzoni-Putman et al., 2021). This fundamental hormonal crosstalk is further modulated by gibberellins (GA), promoting elongation, and abscisic acid (ABA), which induces dormancy under stress (Müller and Leyser, 2011). At the molecular level, key transcription factors such as Shoot Meristemless (STM), responsible for maintaining meristem identity, together with components of the More Axillary Shoots (MAX) pathway (e.g., *MAX1*, *MAX2*, *MAX3*) that regulate auxin signaling, play key roles in bud development (Wang et al., 2018; Cao and Jiao, 2020; Yuan et al., 2024). The regulation is made more complex by microRNAs like miR164, which modulate gene expression post-transcriptionally (Fouracre and Poethig, 2016). Environmental factors, including photoperiod, temperature, and nutrient availability, also significantly affect branching by altering hormonal pathways and gene expression. For example, long-day conditions suppress branching by modifying auxin transport patters, cold temperatures induce bud dormancy, and nitrogen deficiency enhances branching by lowering auxin concentrations (Barbier et al., 2019). Additionally, metabolomic studies have highlighted important biochemical changes associated with bud developmental stages (Zhao et al., 2025). Overall, shoot branching patterns demonstrate considerable phenotypic plasticity, shaped by the integration of environmental signals, genetic factors, and hormonal regulation.

Genome-wide association studies (GWAS) have emerged as a crucial tool for elucidating the genetic basis of complex traits, including shoot branching, a key agronomic characteristic that influences plant architecture, yield, and adaptability to environmental conditions (Susmitha et al., 2023). Through the analysis of dense genetic markers alongside phenotypic variation across diverse populations, GWAS facilitates the identification of significant associated loci (SALs) and candidate genes involved in shoot branching across various crops, such as rapeseed (Li et al., 2016; Zheng et al., 2017), chickpea (Bajaj et al., 2016), soybean (Shim et al., 2019), and cotton (Fu et al., 2019). Recent technological advances, including high-throughput SNP arrays, whole-genome resequencing, and refined phenotyping platforms, have substantially enhanced the resolution and accuracy of GWAS. The use of advanced statistical approaches, including mixed linear models and machine learning algorithms, has further increased detection power while accounting for population structure and confounding effects (Lu et al., 2025). For example, in wheat, GWAS has revealed the role of *VRN1* in regulating shoot-root balance and overall plant architecture (Li et al., 2019). In *Brassica napus*, loci such as *BnaA10g09290D* and *BnaC08g26640D* have been associated with shoot-related traits under low phosphorus-deficient conditions, presenting promising targets for marker-assisted selection to enhance phosphorus use efficiency (Liu et al., 2021). Hormonal signaling pathways also play a crucial role in shoot branching, with GWAS identifying key regulators such as *OsTB1* in rice that influence lateral branching by controlling auxin transport (Takeda et al., 2003). In soybean, GWAS has pinpointed loci associated with determinate and indeterminate branching types, essential for adapting to different cropping systems (Liang et al., 2022). Similarly, in sunflower, GWAS have uncovered several genomic regions associated with distinct branching patterns, showing that apical and basal branching are predominantly governed by distinct genetic loci. These studies also identified multiple candidate genes linked to shoot branching (Baute et al., 2015; Nambeesan et al., 2015). Functional analyses have further emphasized *HaTCP1* as an important negative regulator of shoot branching (Wu et al., 2023). Furthermore, a recent genome-wide study in sunflower identified 12 phosphatidylethanolamine-binding protein (PEBP) genes, particularly FT-like and TFL1-like members, that play crucial roles in regulating flowering and plant architecture (Sun et al., 2025). Collectively, these findings underscore the effectiveness of GWAS in elucidating the genetic basis of shoot branching and its potential for advancing molecular breeding aimed at improving plant architecture and resource use efficiency.

This study presents a GWAS of shoot branching in sunflower utilizing a diverse panel of 82 accessions. Through the integration of GWAS results, Linkage Disequilibrium (LD) block mapping, and transcriptome profiling, the study identified 13 high-confidence candidate genes, including two long non-coding RNAs (lncRNAs). Co-expression analysis revealed potential regulatory interactions between these lncRNAs and several protein-coding candidate genes. To confirm their functional roles, their gene expression patterns across different tissues were analyzed using RNA-seq data and qRT-PCR on contrasting phenotypes. The identified SNPs and candidate genes provide valuable insights into the genetic mechanisms controlling shoot branching and present promising targets for marker-assisted breeding to improve sunflower plant architecture.

## Materials and methods

### Plant materials and growth conditions

The genetic basis of shoot branching in sunflower was investigated using a panel of 82 cultivated sunflower accessions exhibiting diverse branching phenotype (**Supplementary Table S1**). These accessions were cultivated under field conditions at the Yuanyang Experimental Station of Henan Academy of Agricultural Sciences, Zhengzhou, China (35°05′ N, 113°97′ E). Each accession was planted with six biological replicates in insect-proof net houses with 40cm spacing between rows and 20cm between individual plants. Young leaf tissues were collected at the seedling stage for genomic DNA extraction and subsequent resequencing. The branching traits were evaluated at the flowering stage using a standardized phenotypic classification system.

For transcriptome analysis, two representative inbred sunflower lines with contrasting branching phenotype were selected: the single-stemmed line SH18 and the multi-stemmed line MH15. These plants were grown in 5×5 cm pots under controlled greenhouse conditions (16-h light/8-h dark photoperiod, 300 μmol·m^-2^·s^-1^ light intensity, 20 ± 2 °C temperature, and 60% relative humidity). Shoot apical meristem (SAM) tissues were harvested at the second true-leaf stage (approximately 35d post-germination). For each line, SAMs from three individual plants were combined to form one biological replicate, with three replicates per genotype. All samples were immediately frozen in liquid nitrogen and stored at –80 °C until RNA extraction.

### Phenotypic evaluation of branching

Branching traits were evaluated at the flowering stage in field-grown plants. To maximize the contrast between extreme phenotypes, we employed a simplified binary classification system. Each accession was categorized as either single-stem (uniculm) or multi-stem (branching) based on the presence or absence of visible axillary branches along the main stem. This binary classification can be utilized in sunflower branching and domestication studies (Blackman et al., 2010; Baute et al., 2015), providing a reliable framework for GWAS and transcriptomic integration.

While this simplified classification does not capture the full continuum of branching variation (e.g., branch number, branch position, or timing of branch initiation), it provides a clear and biologically relevant dichotomy suitable for association and expression analyses. Detailed accession-level phenotype data are provided in **Supplementary Table S1**.

### Anatomical observation of SAM

To characterize the early morphological features associated with AM initiation, SAMs were harvested from single-stem sunflower plants at the seedling stage. The tissues were dissected longitudinally dissected and immediately fixed in 2.5% glutaraldehyde at 4 °C for 2–4 h. Following fixation, the samples were rinsed with phosphate buffer, post-fixed overnight in osmium tetroxide, and subjected to a graded ethanol dehydration series (30% to 100%, 10–15 min per step). Once dehydrated, the samples were dried using a critical point dryer with liquid CO_2_ (HCP-2, Hitachi, Japan), mounted on aluminum stubs, sputter-coated with gold, and imaged using a Hitachi TM-1000 scanning electron microscope (SEM; Hitachi High-Tech Corporation, Japan).

### Population resequencing and variant detection

Genetic variations associated with shoot branching were identified through whole-genome resequencing of the 82 sunflower accessions mentioned earlier. Genomic DNA was extracted from pooled young leaf tissues of four plants per accession using a modified CTAB protocol. DNA quality and integrity were assessed through 1% agarose gel electrophoresis (targeting fragments ≥20 kb) and quantified with a NanoDrop spectrophotometer (A260/A280: 1.8–2.0). Sequencing libraries with 350 bp insert sizes were constructed using the NEBNext^®^ Ultra^™^ II DNA Library Prep Kit (NEB, USA) and sequenced on an Illumina HiSeq 2500 platform (paired-end, 150 bp reads) at Annoroad Gene Technology Corporation (Beijing, China). Raw reads were filtered using fastp (v0.23.4) to remove adapters, low-quality bases, and reads shorter than 20 bp (Chen, 2023). Clean reads were aligned to the reference sunflower genome (*Helianthus annuus*, HanXRQr2.0) using BWA-MEM (v0.7.17) (Li, 2013). PCR duplicates were eliminated with the MarkDuplicates tool from Picard (v2.27.4, http://broadinstitute.github.io/picard). Variant calling was performed using GATK HaplotypeCaller (v4.2.6.1) (van der Auwera and O’Connor, 2020). Raw SNPs were filtered based on the following criteria: QD < 2.0 || MQ < 40.0 || FS > 60.0 || SOR > 3.0 || MQRankSum <−12.5 || ReadPosRankSum < −8.0.

### Genome-wide association analysis

To identify genetic variants linked to shoot branching in sunflower, a GWAS was conducted using SNP data. The association mapping was performed with GEMMA (v0.98.1) employing a linear mixed model (LMM) that accounts for both population structure and relatedness among individuals to minimize false-positive associations (Zhou and Stephens, 2012). Population stratification was evaluated by principal component analysis (PCA), while kinship (K) matrices were estimated within GEMMA and included in the model. Genome-wide association signals were visualized with Manhattan and quantile-quantile (Q-Q) plots generated via the qqman R package to evaluate the model fit (Turner, 2014). A stringent Bonferroni correction was implemented to control for multiple testing, establishing a genome-wide significance threshold of -log_10_(*P*) ≥ 8.33, corresponding to a p-value of 0.05 divided by the total number of SNPs (6.02×10^6^). For transparency, we also compared results using FDR correction, which yielded a similar set of top signals.

### Transcriptomic profiling and data processing

To examine transcriptomic variations associated with shoot branching, SAMs were collected from 20 uniformly grown plants of each sunflower genotype. The samples were grouped into three biological replicates for RNA sequencing. The SAM tissues were immediately flash-frozen in liquid nitrogen and stored at -80 °C until RNA extraction. Total RNA was extracted using TRIzol^®^ Reagent (Invitrogen, Shanghai, China) and treated with DNase I (Thermo Scientific, USA) to eliminate genomic DNA contamination, following the manufacturers’ protocols. RNA quality was evaluated with the Agilent 2100 Bioanalyzer (Agilent Technologies, USA), and only samples with an RNA integrity number (RIN) ≥ 7.0 were selected. RNA concentrations were determined using a NanoDrop 2000 spectrophotometer (Thermo Scientific, USA), and the samples were standardized to 1 μg/μL for library construction.

RNA-seq libraries were prepared using the NEBNext^®^ Ultra^™^ II RNA Library Prep Kit (New England Biolabs, USA) according to the manufacturer’s instructions. Paired-end sequencing (2 × 150 bp) was performed on the Illumina HiSeq 4,000 platform at Novogene (Beijing, China).

Raw sequencing reads were evaluated for quality and trimmed using FastQC (v0.11.3) to eliminate low-quality bases and adapter sequences. Clean reads were aligned to the sunflower reference genome using STAR (v2.7.8) with default settings (Dobin et al., 2013). Transcript abundance was quantified using RSEM (v1.2.28) (Li and Dewey, 2011).

Gene expression levels were normalized as fragments per kilobase of transcript per million mapped reads (FPKM). Differentially expressed genes (DEGs) between genotypes were identified using the edgeR and limma-voom framework (Ritchie et al., 2015). Prior to modeling, genes with counts per million (CPM) ≥ 1 in at least two biological replicates per genotype were retained. Differential expression was tested using a negative-binomial generalized linear model with genotype as the main effect. P-values were adjusted for multiple testing with the Benjamini–Hochberg method, and genes with |log2FC| ≥ 1 and false discovery rate (FDR) < 0.05 were considered significantly differentially expressed. For robustness, additional analyses were conducted using stricter cutoffs (|log2FC| ≥ 1.5, FDR < 0.01). Functional annotation of DEGs was conducted using the clusterProfiler package based on Gene Ontology (GO) terms (Wu et al., 2021), and data visualization was carried out with ggplot2 (Villanueva and Chen, 2019).

### LncRNA co-expression network construction and functional prediction

To investigate the potential regulatory roles of lncRNAs in shoot branching, a co-expression network was constructed linking lncRNAs and mRNAs located within the GWAS-defined LD regions. Two lncRNAs, identified as DEGs, were chosen as hub regulators. Pearson correlation coefficients were calculated between the expression levels (FPKM values) of these lncRNAs and the mRNAs of candidate genes derived from GWAS and transcriptome analyses. Gene pairs were considered to demonstrate potential trans-regulatory interactions if they satisfied the following criteria: absolute correlation coefficient (|r|) > 0.8 and a p-value < 0.05. Significant lncRNA–mRNA pairs were utilized to construct the regulatory interaction network. The network was visualized using Cytoscape (v3.9.1), with lncRNAs functioning as central nodes (Shannon et al., 2003). To determine the biological functions potentially regulated by these lncRNAs, GO enrichment analysis was performed on the co-expressed protein-coding genes using the clusterProfiler R package (v4.4.4) (Wu et al., 2021). Enriched biological processes were deemed significant at a false discovery rate (FDR) < 0.05.

### Real-time quantitative PCR validation

To confirm the expression patterns of the candidate genes, total RNA was extracted from sunflower stem tissues collected from two contrasting germplasm accessions using the Polysaccharide Polyphenol Total RNA Kit (Tiangen, Beijing, China), following the manufacturer’s protocol. The RNA integrity was evaluated by agarose gel electrophoresis and quantified with a Nanodrop spectrophotometer (Thermo Scientific, Waltham, MA, USA). Genomic DNA contamination was removed with DNase I (Thermo Scientific, #EN0521), and first-strand cDNA was synthesized using the RevertAid™ First Strand cDNA Synthesis Kit (Thermo Scientific, #K1622). Quantitative real-time PCR (qRT-PCR) was performed on a LightCycler^®^ 480 II Real-Time PCR System (Roche Diagnostics, Basel, Switzerland) with gene-specific primers designed by Primer Premier 5.0 (http://www.premierbiosoft.com/primerdesign/index.html; accessed 8 January 2021). Each reaction was performed in triplicate. The sunflower *β*-tubulin gene was used as an internal reference, and relative gene expression levels were calculated using the 2^^−ΔΔCt^ method. For statistical analysis, ΔCt values were compared between the two germplasms using a two-tailed unpaired Student’s *t*-test (Welch’s correction applied when variances were unequal). P-values were adjusted for multiple comparisons using the Benjamini–Hochberg false discovery rate (FDR), and FDR < 0.05 was considered significant. Primer sequences are provided in the **Supplementary Table S2**.

## Results

### GWAS for shoot branching trait

To identify the key genetic factors controlling shoot branching in sunflower, whole-genome resequencing was conducted on 82 sunflower accessions, including 21 multi-branched and 61 single-stem lines. The analysis generated 2,653.46 Gb of high-quality clean data with an average sequencing depth exceeding 10× per accession (**Supplementary Table S1**). The clean reads were aligned to the sunflower reference genome (*Helianthus annuus*, HanXRQr2.0), resulting in the detection of 103,276,072 SNPs distributed across all 17 chromosomes (**Supplementary Table S3**). SNP density differed among chromosomes, with chromosome 11 showing the highest density and chromosome 14 the lowest, averaging 34 SNPs per kilobase (kb) across the genome.

Principal component analysis (PCA) revealed no clear subgroups within the association panel, with the first three PCs explaining only a small proportion of the total genetic variance (**Supplementary Figure S1**). This indicates limited population stratification among the 82 accessions. In addition, the quantile–quantile (Q–Q) plot showed that the observed test statistics closely followed the expected distribution under the null hypothesis, with deviation only at the extreme tail corresponding to significant associations. These results support the robustness of the linear mixed model (LMM) used in the GWAS.

**Figure 1:**
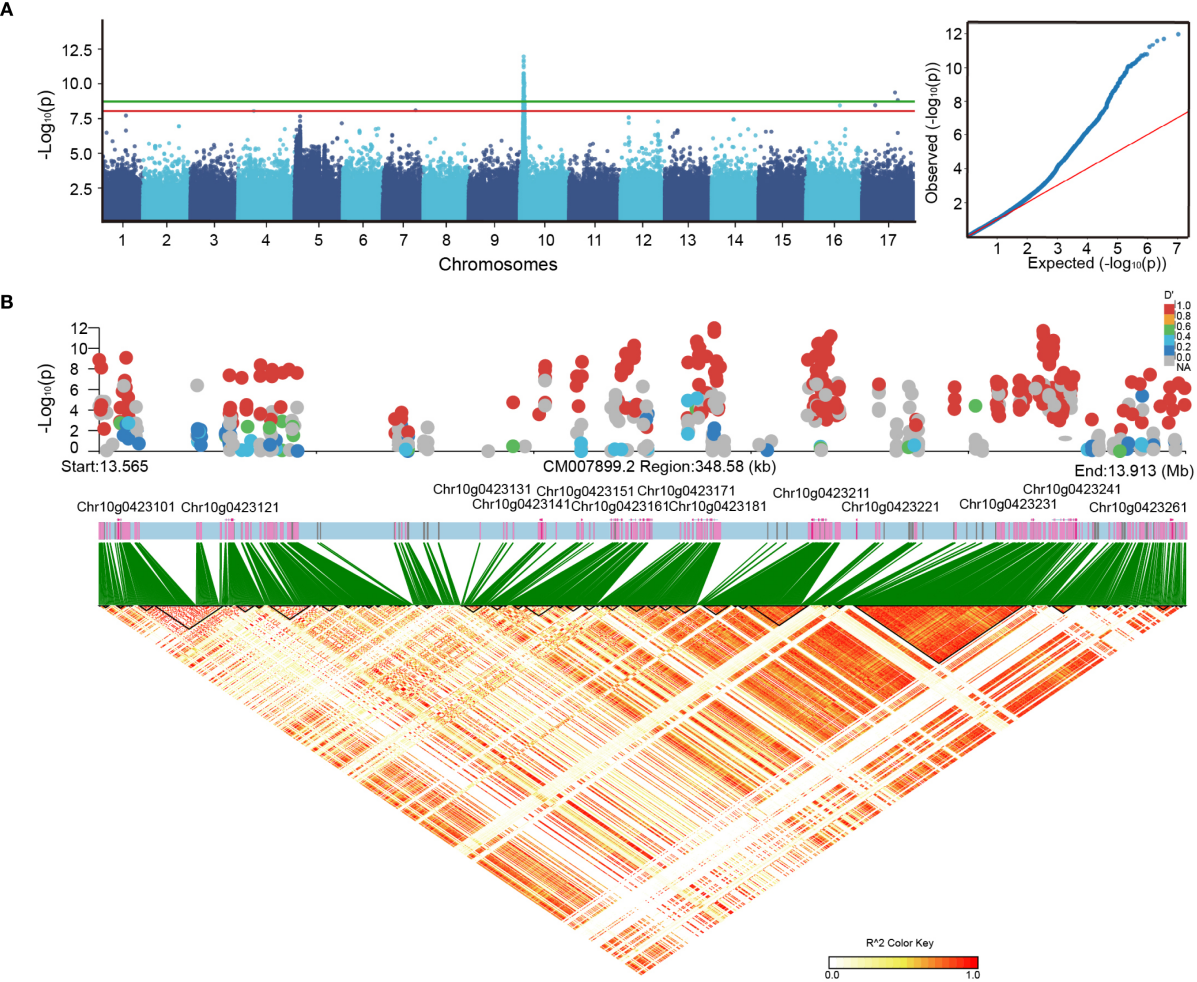
Genome-wide association studies (GWAS) for the branching trait based on single nucleotide polymorphisms (SNPs) and insertions/deletions (InDels). **(A)** Manhattan plot and quantile–quantile (Q–Q) plot showing the significance of associations across the genome. The horizontal line indicates the genome-wide significance threshold. **(B)** Linkage disequilibrium (LD) heatmap surrounding the lead SNP associated with axillary bud development, illustrating the local LD structure.

To identify genetic loci associated with shoot branching, a GWAS was conducted using the GEMMA software with an LMM, applying a stringent significance threshold of p-value < 1.9 x 10^–9^ for SNP-trait associations (**Figure 1A**). This analysis uncovered 62 SNPs significantly associated with the shoot branching phenotype. LD block analysis showed that the majority of peak SNPs (60 out of 62) were concentrated within a 12.40-17.13 Mb interval on chromosome 10 (**Figure 1B**), indicating a potential genetic regulation hotspot. Within this LD region, 113 genes were identified as initial candidate genes potentially involved in regulating shoot branching (**Supplementary Table S4**). For transparency, GWAS results based on false discovery rate (FDR) correction are also provided in the Supplementary Information (**Supplementary Figure S2**), showing consistent signals on chromosome 10.

### Phenotypic characterization of sunflower cultivars with contrasting shoot branching

**Figure 2:**
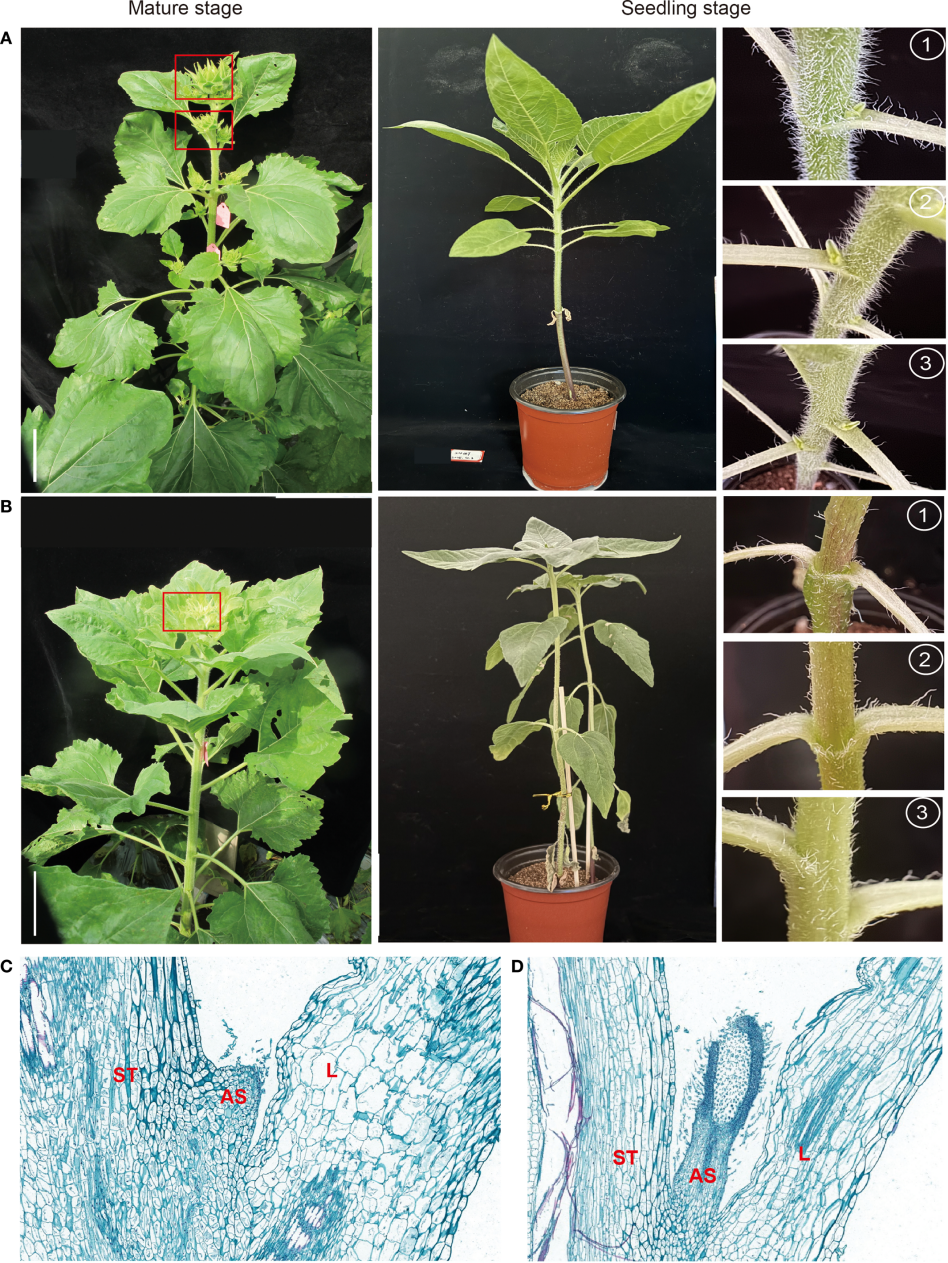
Phenotypic comparison of the multi-stemmed MH15 and single-stemmed SH18 sunflower lines at mature and seedling stages. **(A, B)** Whole-plant phenotypes of MH15 (multi-branched, **A**) and SH18 (single-stem, **B**) at the mature stage (left) and seedling stage (right). The right panels display axillary regions at the first, second, and third pairs of true leaves, illustrating differences in axillary bud development. Red boxes indicate developing capitula. Scale bar = 10cm. **(C, D)** Scanning electron micrographs of median longitudinal sections through the axils of the second **(C)** and third **(D)** true leaves in SH18. AS, axillary shoot; L, leaf; ST, stem.

To explore the developmental basis underlying shoot branching in sunflower, phenotypic characterization was performed on two genetically distinct lines at maturity: the multi-branched cultivar MH15, which produces 3–5 terminal capitula, and the unbranched single-stem cultivar SH18, which forms only one terminal capitula (**Figure 2A**). At the seedling stage (35d after sowing), the visual examination of the axils of the first three true leaves was carried out (**Figure 2B**). Notable phenotypic differences were observed between the two genotypes: MH15 displayed clearly visible axillary buds (0.5–1.2 mm) at all positions, while SH18 exhibited no macroscopic bud structures.

To assess if the absence of visible buds in SH18 resulted from a failure in AM initiation or from a developmental arrest post-initiation, SEM analysis was performed on the second and third true-leaf axils, which are recognized sites of axillary structure initiation in sunflower (**Figure 2C**). SEM results revealed that SH18 did initiate AMs, which appearing as early bulges, but these structures halted development at Stage II and did not develop into leaf primordia (**Figure 2D**). These morphological observations provided a developmental framework for subsequent transcriptomic comparisons between MH15 and SH18 during early branching stages.

### Comparative transcriptome analysis of sunflower genotypes with contrasting shoot branching phenotypes

**Figure 3:**
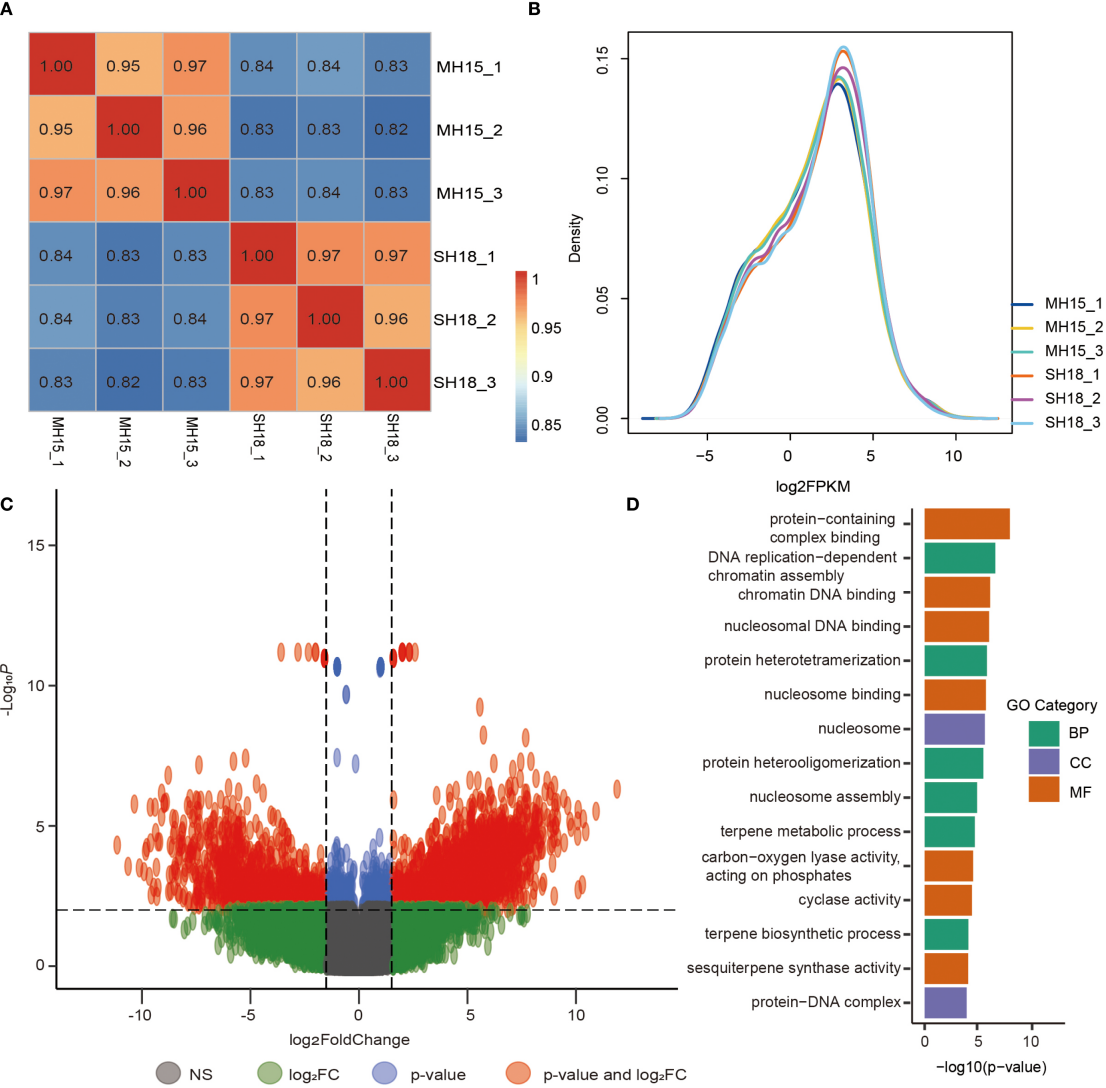
Transcriptomic profiling of shoot apical meristems in sunflower lines with contrasting branching phenotypes. **(A)** Hierarchical clustering of gene expression profiles across three biological replicates for each genotype, showing high consistency and reproducibility. **(B)** Distribution of transcript expression levels (FPKM) across all samples. **(C)** Volcano plot displaying differentially expressed genes (DEGs) between the multi-branched line MH15 and the single-stem line SH18. Gray dots represent non-significant genes (NS), green indicates genes with |log2FC| ≥ threshold only, blue indicates genes significant by adjusted p-value only, and red indicates genes significant for both log2FC and adjusted p-value. The dashed lines denote the cut-off thresholds for log2FC and adjusted p-value. **(D)** Gene Ontology (GO) enrichment analysis of the top 20 enriched terms derived from DEGs, highlighting significantly overrepresented biological processes.

To gain deeper insights into the molecular mechanisms controlling shoot branching in sunflower, transcriptome sequencing was performed on SAM tissues from the multi-branched line MH15 and the single-stem line SH18 at the second true-leaf stage (35-d-old seedlings). After filtering for quality, a total of 40.8 Gb of clean reads data was obtained, with approximately 40,000 transcripts detected per sample. The sequencing quality metrics demonstrated high reliability, with an average Q30 score of 97.25% and mapping rates between 91.33% and 92.55% (**Supplementary Table S5**). Sample clustering confirmed strong consistency among biological replicates, indicating dataset reliability for differential gene expression analysis (**Figure 3A**). Expression level distribution analysis showed that most genes exhibited moderate expression levels (1–32 FPKM) across all samples (**Figure 3B**). Differential expression analysis identified 7,329 genes meeting the threshold of |log2FC| ≥ 1 and FDR < 0.05. A sensitivity analysis using stricter criteria (|log2FC| ≥ 1.5, FDR < 0.01) retained 2,966 DEGs, of which 1,726 were upregulated and 1,204 were downregulated, indicating that the findings were robust to more conservative thresholds. A volcano plot and cumulative DEG counts under varying cutoffs are provided for transparency (**Figure 3C**). GO enrichment analysis (FDR < 0.05, Fisher’s exact test) revealed that these DEGs were significantly enriched in biological processes related to biosynthesis and transcriptional activity, including DNA replication-dependent chromatin assembly, protein heterotetramerizaiton, nucleosome assembly, terpene metabolic process, gene expression, and response to stress (**Figure 3D**). These enriched pathways imply that the transcriptional changes observed may contribute to axillary bud initiation and the developmental variations that lead to the distinct branching patterns seen in sunflower.

### Integration of GWAS and transcriptome analyses reveals candidate genes linked to shoot branching

To validate candidate genes identified through GWAS, an integrative analysis was conducted combining genome-wide association signals, LD block mapping, differential gene expression data, and gene annotations. Among the significant SNPs, 60 were found in non-coding regions (including upstream/downstream regulatory elements, introns, or intergenic regions), while six were located within the coding regions of three genes. Notably, one nonsynonymous SNP (Chr10:13797846, C -> T) was detected in the coding region of *HanXRQr2_Chr10g0423211*, resulting in an amino acid substitution from glutamine (Q) to arginine (R) (**Supplementary Table S6**). This gene encodes an F-box/LRR-repeat protein, which is typically involved in protein degradation and hormone signaling pathways.

Out of 113 genes located in the LD region on chromosome 10, 12 genes overlapped with the 2,996 significant DEGs identified from transcriptomic comparisons between branching and non-branching genotypes. Functional annotations of these 12 genes revealed involvement in hormone signaling, transcription regulation, and meristem development. Notably, two DEGs (*HanXRQr2_Chr10g0422931* and *HanXRQr2_Chr10g0423111*) were identified as lncRNAs, implying a possible regulatory function of non-coding elements in shoot branching. Although *HanXRQr2_Chr10g0423211* did not meet the strict criteria for DEGs, it exhibited moderate but significant downregulation (log_2_Foldchange = -0.45, *p*=0.027), indicating a potential role in branching regulation. Overall, these 13 genes, which include 12 DEGs and one gene harboring a functionally relevant nonsynonymous variant coupled with expression changes, were considered strong candidates associated with shoot branching.

**Figure 4:**
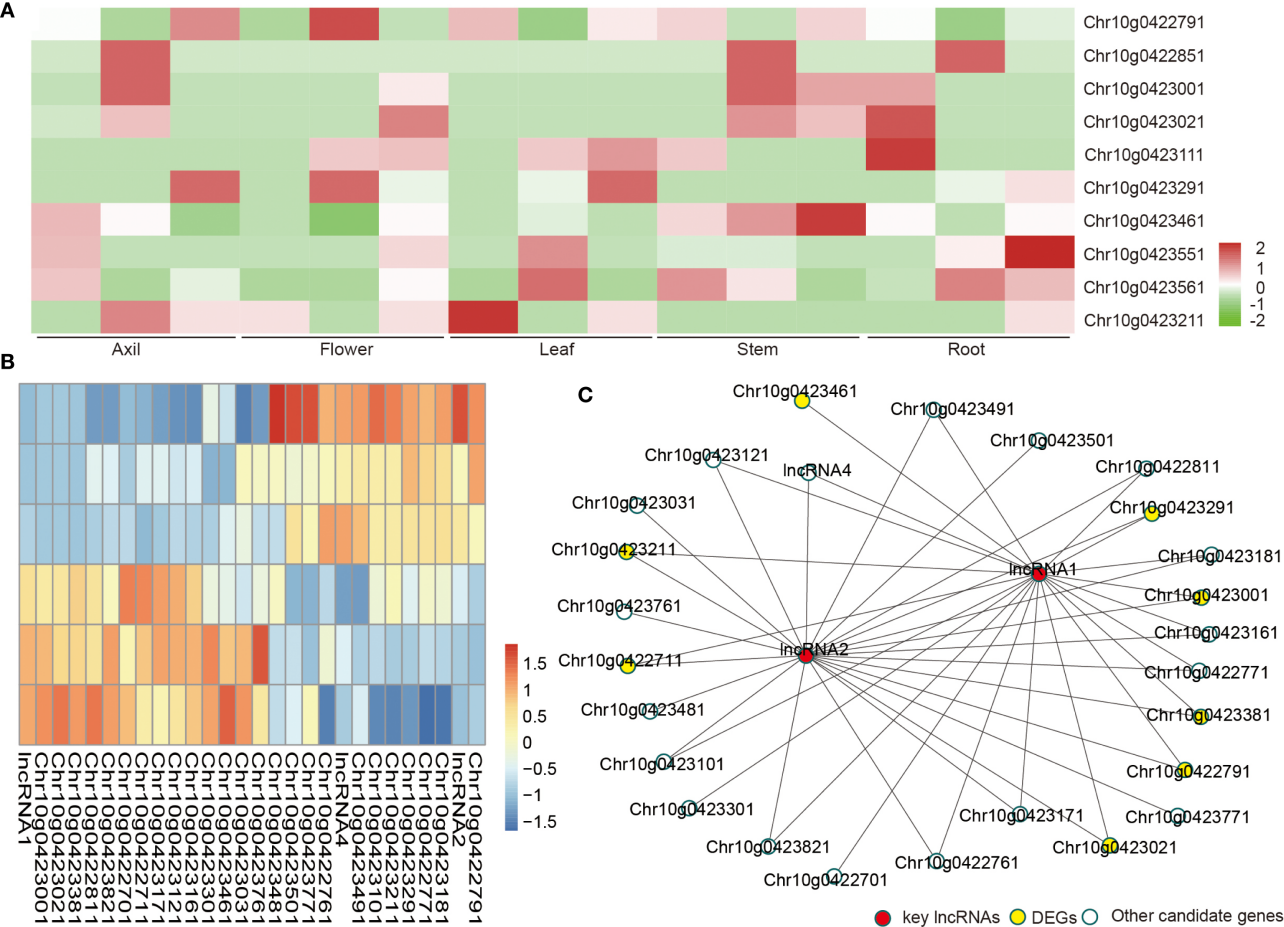
Tissue-specific expression and co-expression analysis of candidate genes and lncRNAs involved in shoot branching regulation in sunflower. **(A)** Heatmap showing the expression profiles of 13 candidate genes across five sunflower tissues (root, stem, leaf, bud, and seed), based on publicly available transcriptome data. **(B)** Co-expression heatmap illustrating the Pearson correlation coefficients between two differentially expressed lncRNAs and the genes within the LD region. High correlation values (|r| > 0.8, *p*<0.05) suggest potential trans-regulatory relationships. **(C)** Co-expression network visualized using Cytoscape, with the two DEG-lncRNAs as central hubs linked to 26 co-expressed genes. Eight of these targets overlap with other GWAS-transcriptome candidate genes, highlighting the potential regulatory role of lncRNAs in axillary bud development.

To further investigate their functional relevance, we analyzed their tissue-specific expression profiles using publicly available transcriptome datasets from five sunflower tissues. Ten of the thirteen candidate genes showed distinct tissue-specific expression, particularly enriched in axillary buds and stem tissues, reinforcing their putative involvement in the regulation of shoot branching (**Figure 4A**).

### Co-expression analysis of long non-coding RNAs

LncRNAs are recognized for their role in regulating gene expression through diverse mechanisms, such as interacting with promoters, enhancers, or transcription regulators. Among the 113 genes located within the GWAS-defined LD blocks on chromosome 10, 14 annotated lncRNAs were identified, along with two microRNAs (miRNAs) and four small nucleolar RNAs (snoRNAs), suggesting the potential role of non-coding RNAs in regulating shoot branching.

To explore the functional significance of the two lncRNAs identified as DEGs, a co-expression analysis was conducted using the FPKM expression values of all 113 genes across samples (**Figure 4B**). Pearson correlation coefficients were calculated between each lncRNA and mRNA pair to detect putative trans-regulatory relationships.

The co-expression network analysis revealed that these two DEG-lncRNAs exhibited significant correlations (|r| > 0.8, p < 0.05) with 26 other genes within the LD interval (**Figure 4C**). Notably, eight of these co-expressed genes overlapped with the remaining 11 candidate genes previously identified through GWAS and transcriptome integration. This overlap strongly suggests that the two lncRNAs may play a regulatory role in shoot branching.

### qRT-PCR validation of candidate genes in stem tissues of sunflower

**Figure 5:**
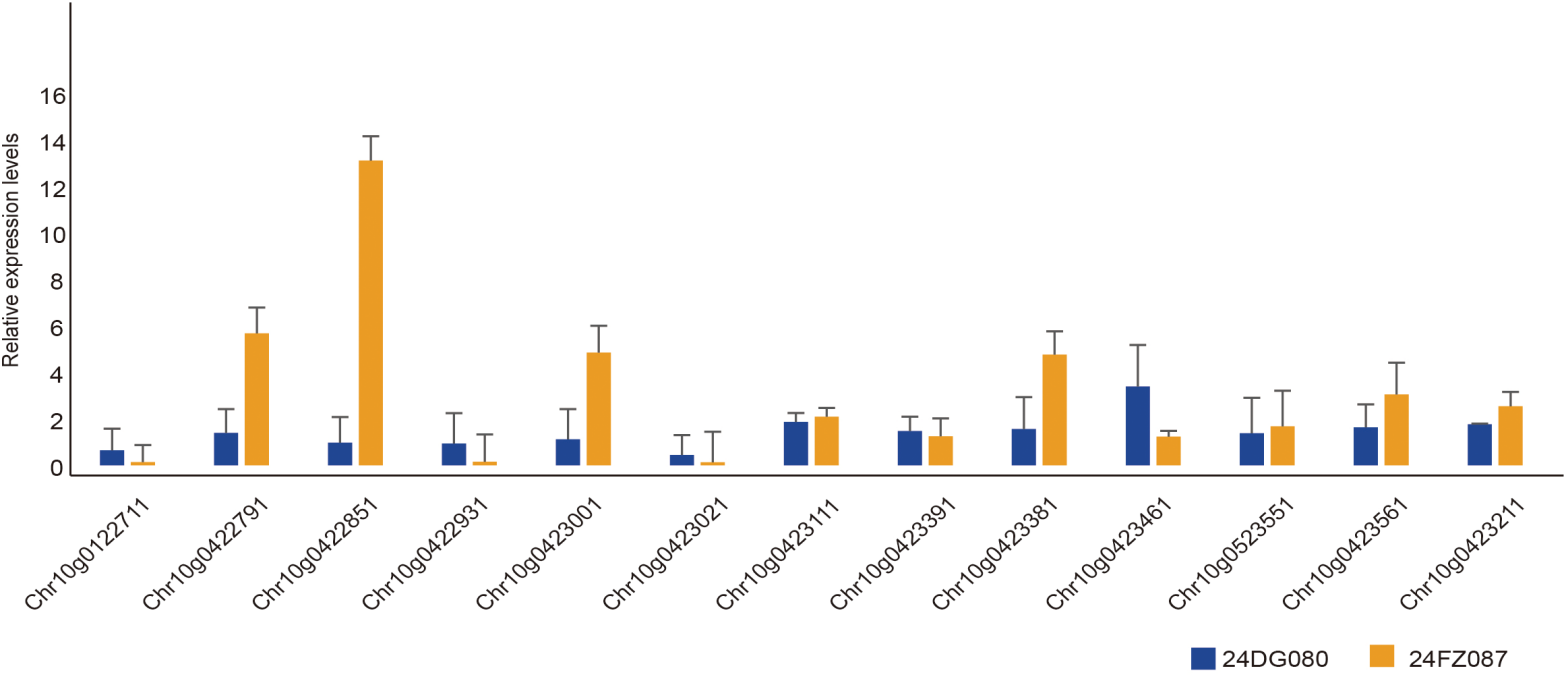
Relative expression levels of 13 candidate genes in the two sunflower varieties. Bars show mean ± SE (n = 3 biological replicates). Statistics: two-tailed unpaired *t*-test on ΔCt (Welch’s correction applied when needed); Benjamini–Hochberg adjustment across genes; *P*<0.05 after FDR considered significant. The germplasm accessions are indicated by color: orange for 24FZ087 (multi-stem) and blue for 24DG080 (single-stem).

To validate the expression patterns of the 13 candidate genes identified through GWAS and transcriptome analysis, qRT-PCR was performed using stem tissues from two contrasting sunflower germplasms: the single-stem type (24DG080) and the multi-stem type (24FZ087). The qRT-PCR results demonstrated substantial concordance with the RNA-seq data, confirming the reliability of the integrative analysis (**Figure 5**). Of the 13 genes, seven showed significantly elevated expression levels in the multi-stem genotype compared to the single-stem genotype (e.g., *HanXRQr2_Chr10g0422851*, *HanXRQr2_0422791*), while three genes showed the reverse trend (e.g., *HanXRQr2_Chr10g0422931*, *HanXRQr2_Chr10g 0122711*). Statistical evaluation of ΔCt values using two-tailed Student’s *t*-tests (adjusted *p*<0.05) confirmed the significance of these genotype-dependent differences. Importantly, two differentially expressed lncRNAs were also verified by qRT-PCR: *HanXRQr2_Chr10g0422931* (lncRNA1) showed higher expression in the single-stem genotype, whereas *HanXRQr2_Chr10g0423111* (lncRNA2) exhibited strong expression in both genotypes, with overall higher levels than lncRNA1. These observations further support the potential regulatory role of lncRNAs in shoot branching. The remaining two genes were expressed in both genotypes but displayed no significant expression differences. Collectively, these findings suggest that genotype-dependent gene expression in stem tissue may contribute to the regulation of shoot branching. Given that axillary meristems are anatomically initiated within stem nodes, stem tissues provide a biologically relevant context for validating candidate branching-related genes.

## Discussion

Sunflower ranks as the world’s fourth most important oilseed crop, with modern cultivars predominantly developed as single-stem hybrids due to their advantageous agronomic traits such as large capitula, uniform maturation, and high seed yield (Badouin et al., 2017; Adeleke and Babalola, 2020). Conversely, multi-stem (branched) sunflower genotypes exhibit a prolonged flowering period, which can help synchronize flowering during hybrid seed production when utilized as male parents alongside single-stem lines (Bachlava et al., 2010). Therefore, gaining insights into the cytological and molecular mechanisms governing shoot branching architecture is crucial for targeted sunflower breeding.

This study examined two phenotypically contrasting sunflower genotypes from our germplasm resource library: the single-stem line SH18 and the multi-stem line MH15 for comparative morphological and cytological analysis. In MH15, axillary buds were visible at the axils of the first pair of true leaves, indicating early initiation and growth of axillary bud primordia (**Figure 2A**). In contrast, although SH18 initiated axillary bud primordia, these structures failed to develop into visible buds at the same developmental stage (**Figures 2C, D**). Due to experimental constraints, SEM-based anatomical observation was conducted only on SH18. However, the consistent presence of fully developed axillary buds in MH15 at the macroscopic level suggests normal initiation and subsequent outgrowth of AM in this genotype. Overall, these observations suggest that the single-stem phenotype in SH18 is not due to failure in AM initiation but rather a developmental arrest during the bud outgrowth phase, potentially caused by enhanced dormancy or suppression of downstream signals required for bud activation.

### Genetic basis of shoot branching in sunflower

Shoot branching significantly influences plant architecture and crop yield by integrating hormonal, sugar, and environmental signals to regulate adaptive growth strategies (Barbier et al., 2019). In the present study, we combined GWAS with transcriptomic analysis across contrasting sunflower genotypes to uncover the genetic factors controlling shoot branching (**Figure 1A**). Although PCA revealed limited population stratification, the inclusion of the kinship matrix in the LMM, together with the Q–Q plot assessment, supports the robustness of our association signals (**Supplementary Figure S1**). The analysis revealed a significant LD block on chromosome 10, encompassing 113 genes associated with the branching phenotype (**Figure 1B**). This region coincides with previously identified quantitative trait loci (QTLs) for branching in sunflower (Baute et al., 2015), underscoring its biological significance. Within this region, *HanXRQr2_Chr10g0423241*, corresponding to *HaGNAT*—a gene previously confirmed through wild-species introgression for its role in axillary bud development—was identified within the LD-defined interval. Although not differentially expressed in our dataset (log_2_FC = 0.06; *p*=0.66), it lies adjacent (only two genes away) to *HanXRQr2_Chr10g0423211*, a primary GWAS candidate harboring a nonsynonymous SNP within an F-box/LRR domain. This gene encodes a putative F-box/LRR protein, a structural class essential to SCF-type E3 ubiquitin ligase complexes that facilitate hormonal signal transduction through protein degradation. Importantly, *MAX2*, a canonical F-box/LRR gene in *Arabidopsis*, governs strigolactone signaling and inhibits shoot branching; *max2* mutants exhibit enhanced branching (Stirnberg et al., 2002; Nelson et al., 2011). Homologous genes in cotton (*GhMAX2*) also demonstrate conserved functions (Peng et al., 2022). Therefore, the proximity of *HaGNAT* to *HanXRQr2_Chr10g0423211* strengthens the significance of this genomic region as a regulatory hub for shoot branching.

This convergence not only validates the reliability of our GWAS results but also highlights chromosome 10 as a genetic hotspot for regulating shoot branching in sunflower. Furthermore, the spatial clustering of functionally relevant loci indicates potential cis-regulatory interactions or common upstream regulatory mechanisms that merit additional functional investigation. In addition, although we adopted the conservative Bonferroni correction to minimize false positives, supplementary analyses using an FDR correction consistently identified the same major association signal on chromosome 10 (**Supplementary Figure S2**), further reinforcing the robustness and biological relevance of this locus.

### Functional transcriptomic insights into axillary bud

Although our integrated genomic analysis identified a strong candidate region on chromosome 10 associated with shoot branching, understanding the molecular mechanisms controlling axillary bud activity remains crucial. To explore the underlying regulatory landscape, we performed transcriptomic profiling of the SAMs from contrasting sunflower genotypes (SH18 and MH15) at the second true-leaf stage to identify gene expression patterns potentially responsible for axillary bud arrest or activation (**Figure 3**). Shoot branching is a complex developmental process requiring both AM initiation and the subsequent lateral bud outgrowth. Our cytological observations demonstrated that both genotypes initiated AMs; however, only MH15 developed visible lateral buds, while SH18 exhibited post-initiation arrest, indicating a transcriptionally mediated dormancy mechanism.

It is worth noting that the number of DEGs identified was highly dependent on the statistical thresholds applied. Using the standard threshold (|log2FC| ≥ 1, FDR < 0.05), we detected 7,329 DEGs, which provided a comprehensive overview of transcriptional differences between MH15 and SH18. Given that such thresholds may still include potential false positives, we further applied a more stringent cutoff (|log2FC| ≥ 1.5, FDR < 0.01), which reduced the number of DEGs to 2,966 (**Figure 3C**). Importantly, functional enrichment analyses based on both sets of DEGs consistently highlighted pathways associated with chromatin assembly, transcriptional regulation, and stress responses, indicating that our biological conclusions are robust across varying stringency levels. This sensitivity analysis demonstrates that the observed transcriptional changes are not artifacts of threshold choice, but rather reflect genuine differences contributing to sunflower branching regulation. GO enrichment analysis identified significant overrepresentation of terms such as macromolecule biosynthetic process, gene expression, and response to hormone, indicating extensive transcriptional and hormonal regulation (**Supplementary Table S4**).

Several candidate DEGs exhibited functional associations, either directly or indirectly, with established regulators of shoot branching (**Supplementary Table S7**). For example, *HanXRQr2_Chr10g0422711*, encoding a ribosomal protein, demonstrated differential expression within the GWAS-defined LD block. Its reduced expression in the single-stem genotype potentially indicates repression by BRC1, a transcriptional repressor that integrates hormonal and environmental signals to maintain bud dormancy (Domagalska and Leyser, 2011; Dun et al., 2012). In *Arabidopsis*, *BRC1* inhibits ribosome-related genes under inhibitory conditions (Yao and Finlayson, 2015), indicating that ribosome biogenesis could be a downstream target in bud arrest. Although vesicle trafficking proteins do not directly regulate branching-related gene expression, they act as key executors within the branching regulatory network by modulating auxin transport and signaling (Gu et al., 2020). The study also identified *HanXRQr2_Chr10g0422791*, encoding the vesicle trafficking protein USE1, which although not previously implicated in shoot branching, is part of the SNARE complex that controls the localization and recycling of auxin transporters like PIN1 and AUX1 (Cui et al., 2022; Luo et al., 2022). Considering the essential role of auxin flux in bud growth, USE1 might indirectly regulate auxin distribution and thus affect bud activity. Chromatin-level regulation was indicated by the differential expression of HanXRQr2_Chr10g0423291, annotated as a histone H2A. In pea, H2A expression increases during axillary bud activation, and variants like H2A.Z influence chromatin accessibility at hormone-responsive loci (Müller and Leyser, 2011; Long et al., 2023). The presence of such a chromatin regulator among the DEGs indicates a potential epigenetic component in the bud arrest observed in SH18. In addition, *HanXRQr2_Chr10g0423551*, a DEG encoding a calcium-transporting ATPase, may affect shoot branching through calcium-mediated signaling pathways that interact with hormonal responses. While not a traditional branching gene, Ca^2+^-ATPases such as ACA8 and ACA10 influence auxin and brassinosteroid signaling by interacting with receptors like BRI1 and CLV1, affecting developmental decisions (Ghosh et al., 2021).

Although canonical dormancy regulators such as *BRC1* or *NCED3* were not among the most significant DEGs, our analysis revealed several genes involved in hormone signaling and transcriptional regulation, potentially contributing to shoot branching. Notably, multiple auxin-related genes exhibited differential expression, including *HanXRQr2_Chr10g0423231*, an AUX/IAA family member, which likely suppresses auxin-responsive transcription and inhibits bud activation (Mazzoni-Putman et al., 2021). Additionally, *HanXRQr2_Chr10g0423211*, a putative F-box/LRR gene described previously, may regulate strigolactone or auxin signaling through SCF-mediated proteolysis (Stirnberg et al., 2002, 2007; Nelson et al., 2011).

Several transcriptional regulators also showed altered expressions. For instance, *HanXRQr2_Chr10g0422811*, annotated as a transcription coactivator of the SSXT family, may participate in cell expansion processes linked to lateral bud outgrowth, as previously observed in leaves and cotyledons (Farrás et al., 2001). Other differentially expressed transcription factors include *HanXRQr2_Chr10g0422801* (C2H2 zinc finger family), *HanXRQr2_Chr10g0423121* (WRKY1), *HanXRQr2_Chr10g0423061* (CCHC-type zinc finger protein), and *HanXRQr2_Chr10g0423031* (bHLH family). These regulators are known to participate in hormonal crosstalk and developmental transitions, potentially shaping the transcriptional landscape governing axillary bud arrest (González-Grandío et al., 2013; Wang et al., 2018; Barbier et al., 2021; Xiong et al., 2024).

Overall, our results indicate a coordinated transcriptional network involving auxin, CK, ABA, and strigolactone-responsive elements that likely maintain bud dormancy in SH18. The differential expression of these signaling components and transcriptional regulators supports the hypothesis that axillary bud arrest results from inhibitory signals that suppress further bud growth rather than the absence of meristem initiation.

### Potential regulatory roles of long non-coding RNAs in shoot branching

LncRNAs have emerged as important regulators of plant development, operating through diverse mechanisms such as chromatin remodeling, transcriptional interference, and modulation of mRNA stability or translation (Zhang et al., 2012; Zhu et al., 2013). Recent studies have increasingly emphasized their involvement in meristem development and branching architecture in recent years (Zhang et al., 2014; Zhao et al., 2018).

In this study, two differentially expressed lncRNAs (*HanXRQr2_Chr10g0422931* and *HanXRQr2_Chr10g0423111*) were identified within the GWAS-defined LD block on chromosome 10. Co-expression network analysis revealed that these two lncRNAs exhibited significant correlations with 26 genes in the LD region, including eight that overlapped with the top GWAS-DEG candidate genes (**Figures 4B, C**). Their strong co-expression with multiple branching-related candidate genes suggests a potential trans-regulatory role in controlling axillary bud development. The correlated genes encompassed transcription factors and signaling components, potentially involved in hormone signaling and meristem fate determination. Intriguingly, one lncRNA (*HanXRQr2_Chr10g0422931*) exhibited higher expression in the single-stem genotype, suggesting a possible role in repressing lateral bud outgrowth. The other lncRNA (*HanXRQr2_Chr10g0423111*) showed high expression across both genotypes, indicating a potential broader role in developmental regulation rather than branching specificity.

Evidence from other species further substantiates the potential of lncRNAs as key regulators of branching architecture. In *Arabidopsis*, the lncRNA *ASCO* interacts with nuclear speckle RNA-binding proteins to modulate alternative splicing of auxin-responsive genes, thereby influencing axillary bud activation (Zhao et al., 2018). In rice (*Oryza sativa*), *LDMAR* (Long-Day–Specific Male Sterility–Associated lncRNA) not only regulates male fertility but is also positively linked to panicle branching under long-day conditions (Ding et al., 2012). Similarly, in maize (*Zea mays*), *TBLR1* (previously lncRNA1459) functions as a competing endogenous RNA (ceRNA) for miR156, promoting the expression of *SPL* transcription factors that enhance tiller and ear branching (Li et al., 2018). These examples illustrate that lncRNAs utilize diverse molecular mechanisms to regulate branching. The conserved functional roles across species provide compelling support for the hypothesis that lncRNAs identified in sunflower may also contribute to the transcriptional regulation of axillary bud development.

One limitation of this study is that the GWAS panel consisted of only 82 sunflower accessions, which is relatively modest given the high genetic diversity of this species. This limited sample size may reduce the statistical power to detect loci with small effect sizes and increase the likelihood of false negatives, while also restricting our ability to capture rare variants. In addition, while Bonferroni correction provides a conservative threshold for minimizing false positives, it may also increase the risk of false negatives, potentially overlooking loci with moderate or rare effects. Despite these limitations, the robustness of our findings is supported by the convergence of multiple lines of evidence: the major association signal was consistently detected across both SNP and InDel datasets, the LD analysis defined a clear 4.7-Mb interval, and transcriptomic profiling further refined the list of biologically relevant candidate genes. Future work with larger and more diverse association panels, combined with high-density resequencing data and complementary statistical approaches, will improve the resolution of causal loci and enable the detection of additional variants contributing to branching regulation.

To validate the RNA-seq findings, qRT-PCR analysis was performed on selected candidate genes using stem tissues from contrasting genotypes (**Figure 5**). The expression patterns were largely consistent with the transcriptomic data, with several genes (including the two lncRNAs) exhibiting genotype-dependent expression. These results reinforce the reliability of our candidate gene set and highlight their potential roles in regulating shoot branching. Although qRT-PCR validation was performed in stem tissues rather than axillary meristems due to the technical difficulty of isolating sufficient RNA from small meristematic regions in sunflower seedlings, the observed expression patterns were consistent with transcriptome data. The use of stem tissues adjacent to the branching sites as a proxy has also been adopted in other studies investigating branching regulators in model and crop species (Aguilar-Martínez et al., 2007; Jiao et al., 2010; Wang et al., 2015). Future studies using meristem-targeted approaches such as laser-capture microdissection or single-cell RNA-seq will provide more direct validation of meristem-specific expression.

It should also be noted that our branching phenotyping relied on a simplified binary classification (single-stem *vs*. multi-stem) at the flowering stage. While this approach effectively captured the major architectural contrast between extreme types, it did not account for the continuous variation in branch number, position, or developmental timing that exists across sunflower germplasm. Such a binary classification has been employed in previous sunflower branching and domestication studies (Blackman et al., 2010; Baute et al., 2015), where it provided a reliable framework for GWAS and transcriptomic integration. However, this approach may have overlooked certain subtle phenotypic differences in our GWAS framework. Future studies integrating quantitative trait scoring across larger and more diverse populations will be essential to refine the genetic architecture of branching and fully dissect the continuum of branching variation in sunflower.

## Conclusion

This study integrated GWAS, transcriptome profiling, and gene expression validation to identify candidate genes involved in shoot branching regulation in sunflower. The analysis revealed 13 potential genes, including two lncRNAs, within a major LD block on chromosome 10. These findings provide novel insights into the genetic basis of branching architecture and offer valuable targets for molecular breeding to improve plant architecture in sunflower.

## Data Availability

The whole-genome resequencing data of the sunflower accessions used in this study have been deposited in the National Genomics Data Center (NGDC) under BioProject PRJCA045124. The raw RNA-seq data generated from the same accessions are available in NGDC under BioProject PRJCA045123. Accession numbers corresponding to individual samples are provided in **Supplementary Tables S1** and **S5**.
