# Supplementary material for: Integrative GWAS and transcriptomic analyses reveal regulatory genes controlling shoot branching in sunflower
Source: Front Plant Sci. 2025 Sep 22;16:1674383. doi: 10.3389/fpls.2025.1674383 (PMC12497784; doi:10.3389/fpls.2025.1674383)
Supplement: Supplementary file 1 [file DataSheet1.docx]

**Integrative GWAS and Transcriptomic Analyses Reveal Regulatory Genes Controlling Shoot Branching in Sunflower**

**Yiyi Sun^1^, Yanwen Wang^1^, Jingyan Bai^1^, Jiatong Guo^1^, Guiting Li ^2, 3, 4^, Xiaojie Yang^2, 5^, Qiuzhen Tian ^2, 3, 4^, Yingying Huang^2, 3, 4^, Shuping LV ^2, 5^, Hengchun Cao^2, 3, 4*^, Lingyun Liu^1*^**

^1^ School of Life Sciences, Henan University, Kaifeng, Henan, 475001, China

^2^ Henan Key Laboratory of Specific Crops Genomics (Henan Sesame Research Center, Henan Academy of Agricultural Sciences), Zhengzhou, Henan, 450002, China

^3^ Henan Joint Key Laboratory of Specific Oilseed Crops, Zhengzhou, Henan, 450002, China

^4^ Henan Sesame Research Center, Henan Academy of Agricultural Sciences, Zhengzhou, Henan, 450002, China

^5^ Economic Crop Research Institute, Henan Academy of Agricultural Sciences, Zhengzhou, Henan, 450002, China

***Correspondence:** chczhima@163.com (H.C.) and lingyunl@henu.edu.cn (L.L.)

suimuyinyang01@163.com (Y.S.); wendtydty111@163.com (Y.W.); b2455515414@163.com (J.B.); 2221010075@henu.edu.cn (J.G.); liguiting07111106@163.com (G.L.); tianqiuzhen@163.com (Q.T.); hncotton6356@163.com (S.L.), chczhima@163.com (H.C.); yyxxjj7910@163.com (X.Y.); lingyunl@henu.edu.cn (L.L.)

**Number of supplementary figures/tables: 7**

**Supplementary Legends**

**Fig. S1:** Principal component analysis (PCA) plot showing the distribution of 82 sunflower accessions along the first two principal components (PC1 and PC2). The accessions exhibit limited separation, indicating weak population stratification within the panel.

**Fig. S2:** Manhattan plot and quantile–quantile (Q–Q) plot of GWAS results based on FDR correction. In contrast to the stringent Bonferroni threshold, FDR identifies significant loci at an adjusted p-value < 0.05. Notably, the major association peak on chromosome 10 was consistently detected under both Bonferroni and FDR, confirming the reliability of this genomic region.

**Table S1** Statistics analysis of re-sequencing of 82 sunflower accessions.

**Table S2** Sequences of primers for qRT-PCR.

**Table S3** Summary statistics of SNPs.

**Table S4** Gene features and functional annotations in the 4.7-Mb associated region.

**Table S5** Statistics analysis of transcriptome sequencing.

**Table S6** Information of the screened SNP loci within 4.7-Mb linked interval.

**Table S7** Functional annotation of the 13 differentially expressed genes (DEGs).
