# Supplementary figures and images for: Integrative GWAS and transcriptomic analyses reveal regulatory genes controlling shoot branching in sunflower
Source: Front Plant Sci. 2025 Sep 22;16:1674383. doi: 10.3389/fpls.2025.1674383 (PMC12497784; doi:10.3389/fpls.2025.1674383)

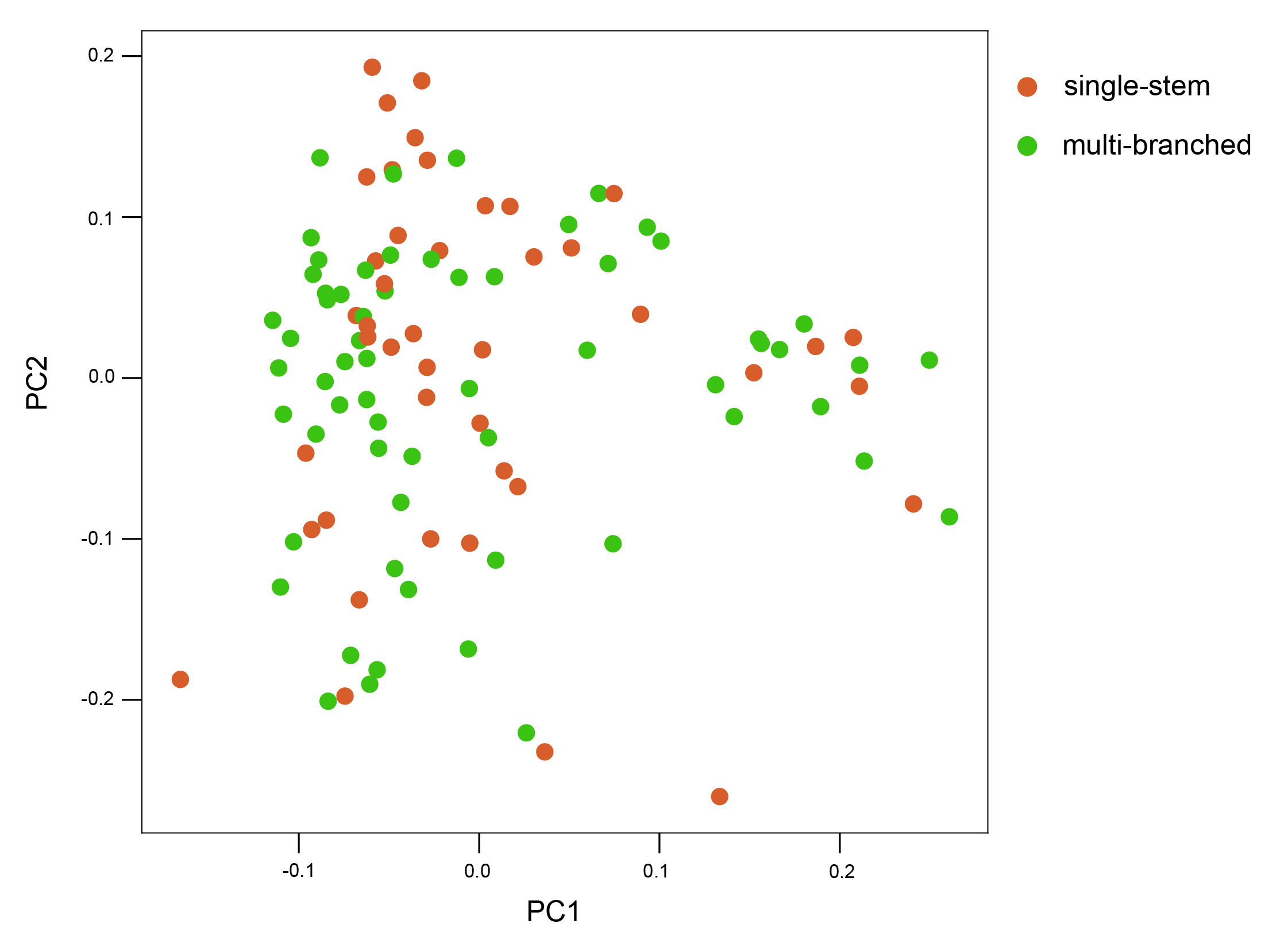

Supplement: Supplementary file 2 [file Image1.jpeg]

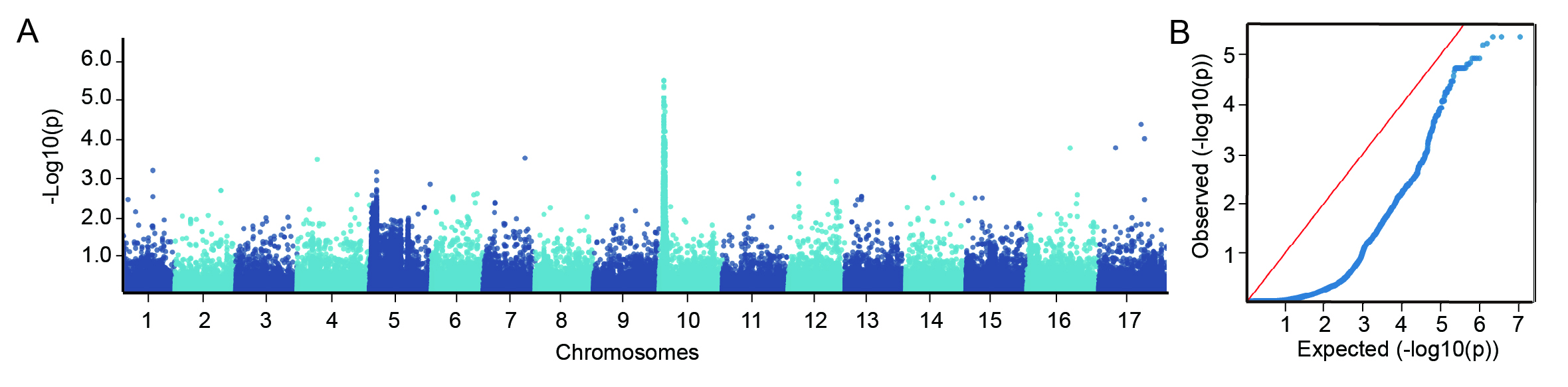

Supplement: Supplementary file 3 [file Image2.jpeg]
