# Supplementary material for: Integrative GWAS and transcriptomic analyses reveal regulatory genes controlling shoot branching in sunflower
Source: Front Plant Sci. 2025 Sep 22;16:1674383. doi: 10.3389/fpls.2025.1674383 (PMC12497784; doi:10.3389/fpls.2025.1674383)
Supplement: Supplementary file 5 [file Table2.docx]

| **Gene ID** | **Primers(5’ -- 3’)** |
| --- | --- |

| **Chr10g0422711** | Forward: TTCTCATAAGGCTGGTTTCT |
| --- | --- |
|  | Reverse: GTTTGTATCTTCCTCGGATT |
| **Chr10g0422791** | Forward: GCTACCACAGGGCGAGTCAA |
|  | Reverse: GACGCAAAGCATTACAAACATCAC |
| **Chr10g0422851** | Forward: TTTCACCGTCATCAAGGTAC |
|  | Reverse: AGGTCCAGCATTATTAGCAG |
| **Chr10g0423001** | Forward: TATTCTCGGATTTCGGTGAT |
|  | Reverse: CTGTCTTCTGCCAACCTCTT |
| **Chr10g0423021** | Forward: TGACCTGCTTGAGCGTGAAA |
|  | Reverse: TGATTTGCTTGGCCCATTTT |
| **Chr10g0423291** | Forward: CTCTGCTGCAATACTGGAAT |
|  | Reverse: CTTGTGAATGTGAGGGATGA |
| **Chr10g0423381** | Forward: TCGGAAGTGGAGGTGGTATG |
|  | Reverse: TACGCACAAATAAAGTTCTTGACG |
| **Chr10g0423461** | Forward: TAAACACCGATTTTGGAGAA |
|  | Reverse: ACTACTGGTTGCGTTTCTGA |
| **Chr10g0423551** | Forward: GTCGTGGCAGTTACAGGTGA |
|  | Reverse: AATACGGTTGAAACAGAGGC |
| **Chr10g0423561** | Forward: TGATTTCAACTCCAGCAGAC |
|  | Reverse: CAAACATTAACCGAAGAAGG |
| **Chr10g0423211** | Forward: CCATTCCGTTGTTCTTTCTC |
|  | Reverse: TGTCACTTATGCTAGACCCACT |
| **lncRNA1** | Forward: ATTGAAATCGGTATCCTTGT |
|  | Reverse: ACTCCGTCATCGCTATAAAC |
| **lncRNA2** | Forward: CTAGAGTTTCGTTAGGGTTT |
|  | Reverse: TCTTGTCTTGTAGCCGTTGA |
| **HaTub** | Forward: CCGTCTTCACTTCTTTATGGTCG |
|  | Reverse: CAACTTCCTTGGTGCTCATCTTT |
